# Tumor Characteristics and Clinical Features of the Patient as Prognostic Factors in PDAC

**DOI:** 10.3390/cancers17203350

**Published:** 2025-10-17

**Authors:** Karina Udrycka, Kamil Rutkowski, Anton Osnytskyy, Ewa Małecka-Wojciesko

**Affiliations:** Department of Digestive Tract Disease, Medical University of Lodz, 90-647 Lodz, Poland; kamil.rutkowski@stud.umed.lodz.pl (K.R.); anton.osnytskyy@stud.umed.lodz.pl (A.O.); ewa.malecka-panas@umed.lodz.pl (E.M.-W.)

**Keywords:** PDAC prognostic factors, pancreatic ductal adenocarcinoma, PDAC staging, grading, metastases, desmoplasia, performance status, nutritional status

## Abstract

Pancreatic ductal adenocarcinoma (PDAC) is the third leading cause of cancer-related deaths among individuals over the age of 50. It is characterized by exceptional aggressiveness and is often diagnosed at an advanced stage, highlighting the importance of assessing prognostic factors. The deep awareness of these factors may help in better prevention and treatment planning, eventually improving the outcomes. Favorable prognostic factors include female gender, low tumor stage, ECOG (Eastern Cooperative Oncology Group) 0–1, lowest ASI (Activated Stroma Index), low-grade tumor budding, age below 40, adequate nutrition, and absence of distant metastases. Conversely, unfavorable prognostic factors include the presence of distant metastases and metastases into lymph nodes, high tumor stage, LVI (lymphovascular invasion), PNI (perineural invasion), tumor size above 3 cm, invasion into vessels, higher G grade, higher ASI, high-grade tumor budding, more than 1 CTC (circulating tumor cells) in the bloodstream, ECOG 3–4, age above 40, Black ethnicity, malnutrition, and sarcopenia. This review discusses the prognostic factors of PDAC related to tumor characteristics and the patient’s clinical issues. The aim of this review is to synthesize current evidence on prognostic determinants in PDAC, with particular attention to both tumor characteristics and patient-specific clinical features. To achieve this, a comprehensive literature review was performed using PubMed, BrowZine Library, Cochrane Library, SpringerLink, Wiley Online Library, BMJ Journals, and Google Scholar. Relevant studies addressing established and emerging prognostic markers were critically analyzed to provide an updated overview of factors that may influence survival and treatment outcomes. By integrating available data, this review seeks not only to summarize classical prognostic variables but also to highlight novel and underrecognized markers that may hold future clinical relevance. Such an approach may contribute to the refinement of prognostic models, support more accurate patient counseling, and ultimately aid in the optimization of therapeutic strategies for individuals affected by PDAC.

## Simple Summary

Pancreatic ductal adenocarcinoma (PDAC) is one of the most aggressive and lethal malignancies, most often diagnosed at an advanced stage when curative treatment is no longer feasible. Despite progress in surgical techniques and systemic therapies, survival gains remain modest. This review moves beyond the conventional TNM framework to explore how both tumor-intrinsic factors—such as histological differentiation, desmoplasia, tumor budding, and the presence of circulating tumor cells—and patient-specific characteristics, including performance status, nutritional condition, and sex-related differences, shape prognosis. By synthesizing established and emerging markers, we provide a more nuanced view of prognostic determinants to support more accurate assessment and stratification of outcomes in patients with PDAC.

## 1. Introduction

Pancreatic ductal adenocarcinoma (PDAC) is the third most common cause of cancer-related deaths among patients over the age of 50. Mortality rates increase by 1% annually, and it has been suggested, that by 2030, PDAC could become the second leading cause of cancer-related deaths [1]. The prognosis for PDAC is highly unfavorable, as evidenced by a 5-year survival rate of only 5–10%, with the median survival time following diagnosis being merely 5–6 months [2,3,4].

PDAC represents 90% of diagnosed pancreatic cancer cases [3]. At early stages, the disease is asymptomatic, or symptoms can be uncharacteristic, due to the deep retroperitoneal location of the pancreas. Consequently, approximately 80% of cases are diagnosed at an advanced stage, with metastatic spread [2,3,5]. Distant metastasis occurs in 50% of cases, most commonly involving the liver, but also the lungs, bones, or peritoneum [2].

Currently, surgical resection is considered to be the most effective treatment, though only 20% of patients qualify for radical surgery [2]. Furthermore, postoperative metastasis and chemotherapy resistance contribute to a high recurrence rate of 80% within two years, and the 5-year survival rate post-surgery is approximately 21% [2,3]. An awareness of prognostic factors may contribute to timely intervention regarding the disease and the optimization of therapeutic strategies.

## 2. PDAC Prognostic Factors Connected with the Tumor

### 2.1. Staging

The higher the stage of PDAC according to the TNM classification, the poorer the patient prognosis, with decreasing chances of effective treatment and shorter survival times. The median survival for patients at stage IA was 29.0–125.9 months, whereas for those in the most advanced stage (IV), it was only 9.0–10.6 months (Table 1) [6,7,8,9].

Although the TNM system is widely used as a prognostic factor, some researchers also highlight its limitations, as it only considers anatomical factors of the tumor (T, N, M). Consequently, patients with resectable tumors may experience recurrence and die within months of surgery, while those with borderline resectable or unresectable disease can achieve long-term survival following surgical resection after neoadjuvant therapy. This is due to the other features of the tumor, such as the level of the biomarkers in the tumor microenvironment, perineural involvement, and the increased biological aggressiveness resulting from its genetic characteristics [10].

Liang et al. [11] propose a postoperative staging system, which incorporates not only the anatomical factors encompassed in TNM but also pathological and biological factors such as perineural invasion (PNI), lymphovascular invasion (LVI), based on the histopathology of the postsurgical specimen and postoperative serum levels of CA19-9 (Carbohydrate Antigen 19-9) and CEA (Carcinoembryonic Antigen) among patients who underwent pancreatectomy. The presence of microscopic LVI is significantly associated with tumor recurrence and poor prognosis in PDAC, while PNI correlates with a high recurrence rate and decreased overall survival (OS) [10]. The OS for patients with PNI was lower than observed for patients without PNI, and yielded 22 and 36 months, respectively [12].

Advancing stage in PDAC almost invariably corresponds with shorter survival, ranging from over a decade in very early disease to less than a year at stage IV. Although the TNM system remains the standard tool, it does not fully reflect the tumor’s biological behavior. Factors such as lymphovascular or perineural invasion and postoperative biomarkers often explain why some patients with resectable disease relapse quickly while others with borderline tumors achieve durable outcomes.

### 2.2. Size of the Tumor

A tumor size greater than 3 cm is significantly associated with poorer prognosis and lower OS usually due to the invasion into surrounding anatomical structures [13,14]. The degree of invasion into vessels such as the superior mesenteric vein (SMV), portal vein (PV), superior mesenteric artery (SMA), celiac artery (CA), and common hepatic artery (CHA) are also critical since they affect the efficacy of the surgical procedures. The longest median survival and the highest 5-year survival rates are observed in patients without vascular invasion, while prognosis worsens with the increasing degree of vascular involvement (Table 2) [14,15].

Larger tumors, particularly those exceeding three centimeters, are more likely to infiltrate nearby vessels and complicate surgical resection. The absence of vascular encasement is associated with the most favorable outcomes, while arterial involvement, even if limited, greatly diminishes long-term survival prospects.

### 2.3. Presence of Metastases in the Lymph Nodes

The N parameter, which describes lymph node involvement, is categorized into stages N0, N1, and N2 based on the number of affected regional lymph nodes [16]. The high N stage is a significant negative predictor of survival for PDAC patients [13].

Valsangkar et al. [11] conducted a retrospective analysis of clinical data from patients with PDAC who underwent surgical resection between 2010 and 2020. Information was collected based on the lymph nodes removed, such as N status (N0/N1), positive lymph nodes (PNL), and the ratio of positive lymph nodes to the total number of examined nodes (LNR), and then the authors determined the optimal number of lymph nodes to be assessed for survival prediction. They found a significant difference in survival between patients with stage N0 and N1 disease, with median survival times of 22 and 14 months, respectively. The study found that the higher number of examined lymph nodes is associated with a higher percentage of patients classified as N1. This suggests that the accuracy of lymph node staging depends on the number of nodes assessed, and examining fewer nodes may lead to misclassification, resulting in N1 patients being classified as N0. They also noted that a higher number of examined lymph nodes (LN) in patients with stage N0 improves the mOS: 16 months for ≤5 LN; 23 months for 6–12 LN; and 28 months for ≥13 LN. This study suggests that in order to accurately determine the extent of lymph node involvement and patient prognosis (nodal staging), it is necessary to evaluate at least 13–16 lymph nodes [11] (Table 3).

Furthermore, some researchers propose that the LNR is also an important prognostic factor [11,12,17]. Pawlik et al. [17], following a thorough analysis of clinical data from patients who underwent pancreatoduodenectomy, demonstrated that with increasing LNR, the mOS decreases: 25 months for LNR = 0; 22 months for LNR > 0–0.2; 15 months for LNR > 0.2–0.4; and 12 months for LNR > 0.4 due to higher disease advancement and cancer progression. The study demonstrated that the number of assessed lymph nodes is a significant prognostic factor, particularly in patients with N0 status. In N0 patients who had fewer than 12 lymph nodes examined, worse prognosis was observed compared to patients who had at least 12 lymph nodes evaluated. This result may stem from an underestimation of disease advancement when the number of lymph nodes assessed is insufficient, which could lead to missed metastases and, consequently, incorrect cancer staging [12,17].

Nodal spread remains one of the strongest adverse prognostic indicators. Both the absolute number of positive nodes and the lymph node ratio have independent value. Importantly, adequate sampling—usually at least 13–16 nodes—ensures reliable staging; otherwise, the disease burden may be underestimated, leading to misleading survival predictions.

### 2.4. Distant Metastases

The liver is the most common site of PDAC metastasis, not only due to its anatomical location but also due to the presence of immune cells such as Natural Killer cells (NK), T cells, Kupffer cells, and non-immune cells like hepatic stellate cells, which promote the formation of a “metastatic niche” (a specific microenvironment within organs that promotes the survival, growth, and development of cancer cells following their migration from the primary tumor) [18]. PDAC metastases can occur at different sites depending on the patient’s age. In older patients, metastases are more frequently observed in the lungs, whereas in younger patients, they are more often found in the liver and other organs, like the bones and brain [5,19]. In case of lung metastases, the median OS is 6–14.6 months. Liver metastases are associated with poor OS due to rich vascular supply with an OS of 4 –11.3 months [18,20,21].

Currently, nomograms are used to predict the risk and prognosis of PDAC metastases to the liver, lungs, and bones. Nomograms allow for the estimation of survival time in patients with metastatic pancreatic cancer and the identification of risk factors for metastasis development, enabling the early recommendation of appropriate diagnostic tests, such as MR (Magnetic Resonance), PET-CT (Positron Emission Tomography) or ECT (Emission Computed Tomography). Early detection of metastases facilitates timely treatment, improving prognosis and quality of life for patients. In the conducted studies, univariate and multivariate logistic regression analyses were employed to identify significant variables, such as age at diagnosis, histological subtype, N stage, presence of metastases, and treatment characteristics, that influence the risk of metastasis. Additionally, using Cox regression analysis, independent prognostic factors were established, allowing for the creation of models to predict survival outcomes. The nomograms, based on the identified variables, present these factors in graphical form, facilitating physicians’ assessment of individual patients’ median survival [2,22,23]. In the conducted study, the predictive model demonstrated a high level of accuracy, as confirmed by a concordance index (C-index) of 0.884 (95% CI: 0.876–0.892) and an area under the ROC curve (AUC) of 0.872. Calibration curve analysis revealed a strong agreement between the predicted and observed outcomes, indicating a precise fit of the model to the clinical data. Furthermore, decision curve analysis (DCA) showed that the model had greater clinical utility compared to the conventional TNM staging system, suggesting improved capability in stratifying patients by risk and providing more accurate support for therapeutic decision-making [2].

One example of a nomogram is the tool developed by Zhang et al. [22], which enables the assessment of the risk of PDAC metastasis to the lungs (PCLM) and the prediction of OS in patients with PCLM. This nomogram incorporates a range of individual clinical characteristics, such as age, tumor stage, tumor size, and histopathological subtype. Based on these factors, the tool predicts individual patient survival at 6, 12, and 18 months. For instance, the probability of survival at 6, 12, and 18 months is 20.7%, 5.5%, and 1.5%, respectively, for a 60-year-old patient with stage II PDAC, a tumor size of 40 mm, no surgical or systemic treatment, and no current metastases to the liver or bones. Compared to the traditional TNM classification system, the nomogram demonstrated significantly higher predictive accuracy, as evidenced by AUC values of 0.871 (95% CI: 0.859–0.883) and 0.884 (95% CI: 0.868–0.900) for the nomogram, versus 0.666 (95% CI: 0.643–0.689) and 0.648 (95% CI: 0.613–0.684) for the TNM system, respectively. The higher AUC values indicate the superior ability of the model to discriminate between patients with different risk levels, suggesting greater utility of the nomogram in predicting clinical outcomes and supporting therapeutic decision-making.

The liver is the predominant target for metastatic colonization, although lung involvement may occasionally be associated with longer survival. Modern nomograms that integrate clinical and pathological parameters provide far better predictive accuracy than TNM alone and are increasingly useful for tailoring follow-up and treatment strategies.

### 2.5. Grading

Patients with well- or moderately differentiated tumors (G1 or G2) had a median survival of 18–22 months, whereas those in the same stage (IIA) but with poorly differentiated or undifferentiated tumors (G3 or G4) had a shorter median survival of 13–19 months. This survival time is comparable to that of patients with more advanced stage IIB disease [8,24]. That is why tumor grade has been incorporated into the TNM classification, leading to the development of the TNMG system. Studies have shown that the TNMG classification, which considers both tumor stage and the differentiation degree, provides greater accuracy in predicting survival for pancreatic cancer patients; for instance, the median survival for a patient in stage IV with G1/G2 differentiation is 15 months, whereas for those with G3/G4 differentiation, it decreases to only 6 months [6,7,8].

The assessment of tumor grade and histological features is critically dependent on the quality of the biopsy specimen, which in turn is strictly influenced by the sampling technique used during EUS-FNB (Endoscopic Ultrasound-Guided Fine-Needle Biopsy). Among the approaches compared are the slow-pull technique (gradual withdrawal of the stylet to create minimal negative pressure), dry-suction (application of a 20 mL negative pressure syringe), modified wet-suction (needle pre-flushed with saline and aspiration using a 10 mL pre-vacuum syringe), and no-suction sampling [25]. A meta-analysis of nine randomized controlled trials (756 patients) demonstrated that the modified wet-suction technique achieved the highest rate of adequate samples (SUCRA 0.90) (Surface Under the Cumulative Ranking Curve), whereas the no-suction approach was significantly inferior (SUCRA 0.14; RR 0.83–0.85 vs. other techniques). Tissue integrity was superior with modified wet-suction (RR 1.36 vs. dry-suction; 95% CI 1.06–1.75; SUCRA 0.89), while the slow-pull method was associated with significantly less blood contamination compared with dry-suction (RR 0.71; 95% CI 0.52–0.97). In terms of diagnostic sensitivity, modified wet-suction yielded the best results (93%), followed by dry-suction (87%) and slow-pull (84%), whereas no-suction achieved only 72% [25]. These findings were corroborated in a multicenter randomized trial including 210 patients, where tissue core procurement was significantly higher with wet-suction compared to slow-pull (71.4% vs. 61.4%, *p* = 0.03; OR 1.58; 95% CI 1.05–2.38). Tissue integrity was also superior with wet-suction (*p* = 0.02; OR 1.66; 95% CI 1.12–2.41), although at the expense of greater blood contamination (*p* < 0.001). Importantly, the proportion of samples with adequate tumor fraction (≥20%) was comparable between the two techniques (84.8% vs. 80.3%; *p* = 0.41), and diagnostic accuracy did not differ significantly [26].

Collectively, these data clearly indicate that reliable histological characterization and accurate tumor grading require not only the use of appropriate biopsy needles but, above all, the application of an optimized and standardized sampling technique [25,26].

Histological grade strongly influences outcome. Poorly differentiated tumors behave aggressively and shorten survival even when diagnosed at lower anatomical stages. The TNMG system, which merges stage and grade, offers a more precise survival estimate than the traditional TNM model.

### 2.6. Other Histopatological Features

#### 2.6.1. Desmoplasia

Desmoplastic reaction and fibrosis are related but distinct pathological processes. Desmoplastic reaction involves the formation of fibrous tissue around a tumor, creating a desmoplastic stroma that isolates the cancer from the surrounding healthy tissues. Fibrosis, on the other hand, is the process of accumulating extracellular matrix (ECM) components, such as hyaluronic acid and collagen, in the interstitial space, leading to tissue stiffness and scarring. Desmoplastic reaction can contribute to fibrosis by creating a stroma that hinders the supply of nutrients and oxygen, promoting the accumulation of ECM and increasing the activity of cancer cells [27].

In PDAC, the tumor is associated with the particularly strong desmoplastic reaction surrounding the primary lesion, which promotes tumorigenesis and angiogenesis. In the process of forming fibrous tissue, cancer-associated fibroblasts (CAFs) play a key role. CAFs are produced by pancreatic stellate cells (PSCs). CAFs upregulate genes involved in extracellular matrix (ECM) synthesis, including SPARC (Secreted Protein Acidic and Rich in Cysteine), whose components, such as type 1 collagen, promote tumor growth and metastasis. Additionally, CAFs activate the CXCL12/CXCR4 (CXC motif chemokine ligand 12/CXC chemokine receptor type 4) chemokine pathway, leading to the induction of epithelial–mesenchymal transition (EMT). This process involves the loss of E-cadherin (an epithelial marker) and the acquisition of vimentin (a mesenchymal marker), resulting in the loss of cell–cell adhesion and a gain in mesenchymal properties, which causes an increased risk of metastasis. What is more, a desmoplastic reaction leads to resistance to chemotherapy, since it leads to the formation of a rigid stroma with dense fibrous tissue that hinders drug penetration into the tumor [16,27,28,29,30].

To assess the tumor microenvironment, the Activated Stroma Index (ASI) was proposed, which reflects the PSC activity and its impact on collagen deposition. ASI is based on the measurement of smooth muscle actin (α-SMA) expression, which is present in activated miofibroblasts produced by PSC. High α-SMA expression indicates increased PSC activity, leading to enhanced desmoplasia. To calculate ASI, α-SMA expression was measured in postsurgical paraffin-embedded tissue samples obtained from patients with histopathologically confirmed PDAC. To assess α-SMA expression, the tissue sections were stained with an α-SMA-specific antibody and aniline blue from Masson’s trichrome staining, which is specific for collagen, allowing for the assessment of collagen fiber presence and distribution within the tumor. Quantitative analysis was then performed using computer-assisted image analysis on digital scans of histological slides. α-SMA-positive staining areas were analyzed in relation to the total tissue area, allowing for the assessment of the density and spatial distribution of activated myofibroblasts within the PDAC microenvironment. The study revealed that patients with the lowest ASI had the best median survival of 25.7 months, while those with the highest ASI had a median survival of only 16.1 months, which confirms the prognostic role of desmoplasia [29].

Fibrosis may also be assessed with endoscopic ultrasound elastography (EUS). This technique allows for the evaluation of tissue stiffness, which aids in distinguishing malignant changes from benign changes and healthy pancreatic parenchyma. Due to the presence of desmoplasia, PDAC exhibits increased tissue stiffness, which in elastography manifests as specific patterns, such as a blue color and a higher strain ratio (a quantitative indicator of tissue stiffness), compared to the surrounding parenchyma. The normal pancreatic parenchyma typically exhibits a homogeneous green pattern with a strain ratio (SR) of 1.68. In the case of inflammatory masses in the pancreas, the pattern is usually heterogeneous, predominantly green, with subtle yellow and red lines, and the SR is 3.28. On the other hand, malignant pancreatic tumors are characterized by a heterogeneous pattern, typically blue, with small green areas and red lines, and the strain ratio is 18.12 [31,32].

The study conducted by García et al. [33] included patients with solid pancreatic masses who underwent endoscopic ultrasound elastography (EUS) to assess the biomechanical properties of these lesions. For the quantitative analysis, two distinct regions were selected within the examination field: region A, which encompassed the largest possible area of the focal lesion (representative of the tumor), and region B, located in the soft (red) reference tissue outside the lesion. The strain ratio (SR) was calculated as the ratio of the values obtained from region B to those from region A (B/A). Final diagnoses were established based on a histopathological examination of surgical specimens and a cytological analysis of samples obtained via EUS-guided fine-needle aspiration (EUS-FNA). The mean SR in healthy pancreatic tissue was 1.68 (95% confidence interval [CI]: 1.59–1.78). In inflammatory masses, this value was significantly higher, averaging 3.28 (95% CI: 2.61–3.96; *p* < 0.001). In PDAC, the mean strain ratio reached 18.12 (95% CI: 16.03–20.21; *p* < 0.001). The highest values were observed in pancreatic neuroendocrine tumors (P-NET), with a mean strain ratio of 52.34 (95% CI: 33.96–70.71), indicating markedly increased stiffness of these lesions. The authors suggest that a strain ratio threshold of >6.04 represents a significant parameter for differentiating pancreatic lesions, allowing for the achievement of a high EUS elastography diagnostic accuracy, reaching 97.7%. Furthermore, elastography enables the detection of solid malignant pancreatic tumors, with the high sensitivity ranging from 92 to 100% and a specificity of 67–92.9% [31,32,33].

In the study conducted by Shi et al. [34] elastographic parameters obtained during EUS were shown to have not only diagnostic but also prognostic relevance in patients with pancreatic cancer. The authors reported that the SR was positively correlated with the proportion of stromal tissue in resected pancreatic cancer specimens (R = 0.768; *p* < 0.001). Higher SR values were associated with poorer overall survival compared with a low SR (15.4 vs. 25.8 months; *p* = 0.017), and multivariate analysis confirmed SR as an independent prognostic factor in resected pancreatic cancer (hazard ratio = 1.939; *p* = 0.020). These findings suggest that assessment of tumor stiffness by elastography may reflect underlying biological characteristics of the tumor that are relevant to patient prognosis.

The dense fibrotic stroma that surrounds PDAC is not merely a bystander but actively drives tumor progression, angiogenesis, and drug resistance. High ASI signals worsen prognosis. Elastography, a non-invasive ultrasound-based tool, can quantify tissue stiffness and may help differentiate PDAC from benign pancreatic lesions with high sensitivity.

#### 2.6.2. Tumor Budding

One of the morphological features of EMT is tumor budding. This process involves individual cancer cells or small clusters of up to five cells detaching from the main tumor mass and being observed at the invasive front of the tumor. It represents an indicator of cancer invasiveness, as the greater the number of ’buddings’, the more invasive the PDAC becomes [9,16]. The evaluation of tumor budding was performed based on a thorough histopathological analysis of PDAC postsurgical specimen where the number and presence of individual cancer cells or small clusters of tumor cells (referred to as ’buddings’) were determined. The study was conducted among patients who underwent resection of PDAC, and the prepared tissue samples were stained using the standard H&E (hematoxylin–eosin) method. The subsequent step involved microscopic analysis to evaluate the number of tumor buds in various areas of the tumor. Two approaches were used to quantify tumor budding. The first involved counting tumor buds in so-called “hot spots” of budding at a 20× magnification, according to the methodology proposed by ITBCC (International Tumor Budding Consensus Conference) in the context of colorectal cancer. Additionally, in areas exhibiting maximum tumor budding, identified through scanning magnification, the number of buds was counted in 10 high-power fields (HPF). Tumor budding was classified into three categories: Bd 1 (0–4 buds), Bd 2 (5–9 buds), and Bd 3 (10 or more buds). Bd 1 and Bd 2 were considered low-grade budding, while Bd 3 was classified as high-grade budding. Poorly budding tumors (Bd 1, Bd 2) were significantly positively correlated with longer DFS (disease-free survival) and OS, which yield 13.1 and 26.7 months, respectively. In contrast, for strongly budding tumors (Bd 3), the median DFS was 6.4 months, and OS was 12.3 months. What is more, researchers demonstrated a correlation with the tumor differentiation grade, because tumors with a grade of G1/2 had an average of 2.74 tumor buds per HPF, while G3 tumors had an average of 7.99 tumor buds per HPF (*p* < 0.001) [35]. Tumor budding has been extensively studied in the context of colorectal cancer and has also been described in adenocarcinomas of the stomach, esophagus, and, among others, in esophageal squamous cell carcinoma. In all these cancers, the presence of tumor budding has been associated with poorer prognosis [36,37,38,39]. Although further research is needed, tumor budding may serve as a negative prognostic factor in PDAC.

The presence of isolated cells or small clusters at the invasive front—referred to as tumor budding—signals epithelial–mesenchymal transition and enhanced metastatic potential. High-grade budding is consistently linked with shorter disease-free and overall survival and is emerging as a useful histological marker in PDAC, as it already is in colorectal cancer.

#### 2.6.3. CTCs—Circulating Tumor Cells

CTCs are the cells originating from the primary tumor or metastases, that enter the bloodstream. Although most of them undergo apoptosis within the first 1 to 2.5 h, some survive and may play a role in metastases development [40]. CTCs circulate throughout the body, often surrounded by platelets, which protect them from the immune system. They get trapped in the capillaries of distant organs, where they undergo extravasation and settle in the tissue, initiating colonization. This process, which can last for years, involves evading the immune response and settling in niches, allowing the cells to survive and initiate the growth of new tumors. After the latency phase, the cells may form the secondary tumors [41].

In a study conducted by Hugenschmidt et al. [42], in patients 5 to 10 years after PDAC resection, the presence of CTCs was found to correlate with shorter disease-free survival (DFS). The detection of circulating tumor cells (CTCs) was performed in patients who underwent PDAC resection using the FDA (Food and Drug Administration)-approved CellSearch^®^ system. Peripheral blood samples were placed into the automated CellTracks Autoprep device, where CTCs were separated from other blood cells using ferrofluidic nanoparticles coated with anti-EpCAM (epithelial cell adhesion molecule) antibodies. CTCs were subsequently immunostained with anti-cytokeratin antibodies (CK8, 18, and 19). In the CellTracks Analyzer II^®^ system, the cells were scanned using fluorescence microscopy. The study demonstrated that the median DFS in patients with detectable CTCs was 3.3 months, whereas in those without CTCs, it was significantly longer at 9.2 months. Moreover, patients with detectable CTCs exhibited shorter cancer-specific survival. The median CSS in this group was 6.6 months, compared to 18.5 months in patients without CTCs.

Furthermore, studies by Court et al. [43] demonstrated a significant relationship between the number of CTCs in peripheral blood and the presence of latent metastases in patients eligible for radical surgery. The number of CTCs was assessed using the NanoVelcro CTC assay which utilizes surfaces coated with nanoparticles, enabling the selective capture of cancer cells from the patient’s blood. These nanostructures are chemically modified to bind to surface proteins of cancer cells, such as EpCAM. Next, the cancer cells were analyzed with fluorescence microscopy, which enables the molecular characterization of CTCs. The study demonstrated that CTCs were detected in 78% of patients with PDAC, and CTC count was positively correlated with advancing disease stage (*p* = 0.42, *p* < 0.001). Detection of one or more CTCs per 4 mL of blood occurred in 44.4% of stage I, 74.2% of stage II, 77.4% of stage III, and 93.1% of stage IV patients. Furthermore, in patients undergoing surgery, using a cutoff of three or more CTCs per 4 mL of venous blood, preoperative differentiation was established between individuals with and without the occult metastases. This method achieved a sensitivity of 85% and specificity of 80%, with an area under the ROC curve (AUROC) of 0.82 (95% CI: 0.76–0.98; *p* < 0.0001). The number of CTCs was also identified as an independent predictor of overall survival (HR 1.38, 95% CI: 1.01–1.88, *p* = 0.040).

CellSearch^®^ is the only FDA-approved system and is relatively widely applied in clinical practice. It is characterized by a high degree of standardization and strong supporting evidence; however, its sensitivity remains limited—CTCs are detected in a smaller proportion of patients, and the results are primarily prognostic in nature (shorter DFS and CSS). In contrast, the NanoVelcro CTC assay employs advanced technological approaches (nanostructures, surface chemical modifications, and fluorescence-based analyses), which increases its complexity and restricts its clinical availability. Nevertheless, this method demonstrates higher sensitivity and specificity, enabling not only the detection of CTCs but also the identification of patients with occult metastases. Evidence suggests that it has greater potential for assessing disease progression and predicting overall survival. In summary, CellSearch^®^ is a standardized and more accessible method, although with lower sensitivity, whereas the NanoVelcro CTC assay is technically more demanding but offers higher accuracy and stronger predictive value in the clinical setting.

## 3. Factors Connected to the Patient

### 3.1. General Condition of the Patient

**Figure 1:**
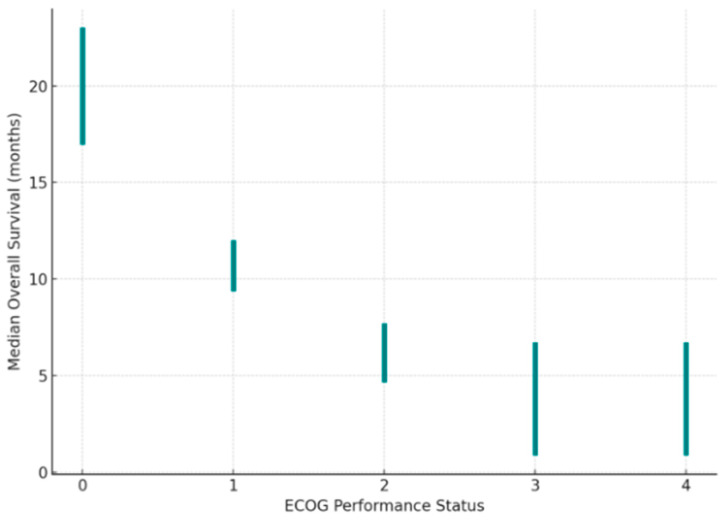
ECOG performance status and median overall survival in PDAC [42,43,44].

Performance status (PS) is a commonly known metric for predicting and determining both medical and surgical therapy options for cancer patients. PS is usually assessed using the Eastern Cooperative Oncology Group (ECOG) scale, which is an indicator of a patient’s overall health status and ability to tolerate cancer therapy [44]. In the context of PDAC, PS plays a crucial role in evaluating a patient’s eligibility for more intensive therapies that may improve their OS [42]. Patients with a higher PS (ECOG 3–4) tend to have a poorer prognosis, shorter survival time, and limited therapeutic options. In contrast, patients with lower PS (ECOG 0–1) are more likely to be eligible for more invasive forms of treatment, such as chemotherapy (Table 4 and Figure 1) [45,46,47].

Nevertheless, it is important to emphasize the subjectivity of the PS assessment, which may lead to divergent results and reduce certain patients’ chances of receiving a more aggressive form of therapy. A more objective approach could be the comprehensive geriatric assessment (GA), recognized by the American Society of Clinical Oncology (ASCO) and the National Comprehensive Cancer Network (NCCN) guidelines as a crucial component of individualized treatment for older cancer patients. Unlike assessments based solely on chronological age and PS, GA provides detailed information on the patient’s functional status, comorbidities, nutritional status, sensory deficits, social support, mental well-being, and cognitive functions. These factors can significantly affect treatment tolerance and outcomes, allowing for more aggressive chemo- and radiotherapy treatment in some cases, when necessary [48].

PS, typically measured by the ECOG scale, continues to be one of the most practical tools for therapeutic decision-making. Patients with ECOG 0–1 usually tolerate aggressive regimens and live longer, while those with ECOG ≥ 3 rarely benefit from intensive treatment. In elderly individuals, comprehensive geriatric assessment offers a more objective perspective than performance status alone.

### 3.2. Age, Race and Sex

#### 3.2.1. Age

Numerous reports indicate that age is a significant negative prognostic factor for pancreatic cancer [5,13]. The significant negative correlation between patient age at the time of PDAC diagnosis and mean survival time has been found (Table 5) [49,50,51].

In addition, in elderly patients, the therapeutic process may be disrupted by the presence of underlying diseases. The study by Tao et al. [5] revealed that patients over 80 years of age with metastatic pancreatic cancer (mPC) exhibited higher overall mortality during the observation period; however, they showed lower CSS. The cancer mortality rates were 82.8% in the group under 65 years of age, 80.8% in the 65–80 group, and 76.8% in patients over 80 years of age. In contrast, for OS, the mortality rates were 91.7%, 95.2%, and 97.1% in the same three age groups, respectively. Elderly patients with PDAC frequently die from causes other than the cancer itself, including cardiovascular diseases, strokes, respiratory disorders, and metabolic conditions with their complications [52]. Comorbidities not only contribute to competing mortality but also complicate oncological management by limiting access to chemotherapy, increasing the risk of adverse events, reducing treatment tolerance, and worsening overall performance status. In clinical practice, the most prevalent comorbidities are cardiovascular diseases (e.g., atrial fibrillation requiring anticoagulation, heart failure, coronary artery disease), diabetes, and chronic obstructive pulmonary disease. In older patients, additional conditions such as renal insufficiency, anemia, and polypharmacy further elevate the risk of bleeding. Importantly, the use of anticoagulants (e.g., low-molecular-weight heparins or direct oral anticoagulants), often indispensable for the prevention and treatment of thrombosis, is associated with a substantially increased risk of major bleeding—reported in 6.5% to 18% of cases—further complicating chemotherapy delivery and therapeutic decision-making [53,54,55].

Table 6 presents a concise summary of the major studies aimed at evaluating the impact of age on the prognosis of patients with PDAC.

Increasing age is associated with progressively shorter survival, particularly beyond 70 years. Comorbidities and frailty often complicate oncological care, not only by limiting chemotherapy but also by contributing to competing causes of death.

#### 3.2.2. Race

In Black patients, the incidence of PDAC is 1.4 times higher than in individuals of other races. The disease is often diagnosed at a younger age and at a more advanced stage in this population [56]. An analysis of risk factors suggests that the higher incidence in this group may be attributed to factors such as higher rates of smoking, diabetes, obesity, and other unfavorable environmental influences. However, the impact of genetic predisposition remains incompletely understood and may also play a significant role in differences in survival rates [57,58].

In the study conducted by Biel et al. [58], transcriptomic analysis was conducted on pancreatic tumor and non-tumor tissues obtained during surgical resection from Black and White patients to identify gene-expression differences potentially contributing to the progression and prognosis of PDAC. RNA-Seq profiling revealed the expression of over 24,900 genes, among which more than 4000 displayed statistically significant differences between tumor and non-tumor tissues. Seven genes—AGR2 (Anterior Gradient 2), CEACAM6 (Carcinoembryonic Antigen-Related Adhesion Molecule 6), GNMT (Glycine N-Methyltranserase), PDIA2 (Protein Disulfide Isomerase Family A Member 2), POSTN (Periostin, Osteoblast Specific Factor), RBPJL (Recombination Signal Binding Protein for Immunoglobulin Kappa J Region-Like), and S100P (S100 Calcium Binding Protein P)— were identified as being specifically overexpressed in tumor tissues regardless of racial background. In the race-based comparison, 1310 genes were found to be differentially expressed between tumors from Black and White patients. Notably, TSPAN8 (Tetrasparin 8) and GSTM1 (Glutathione S-Transferase Mu 1) showed significantly higher expression exclusively in tumors from Black patients. High expression of TSPAN8 was associated with worse overall survival, correlating with a reduction in median survival by 7.5 months and an increase in the hazard ratio to 1.81. Furthermore, within the PD-1/PD-L1 (Programmed Cell Death Protein 1/Programmed Death-Ligand 1) immunoregulatory pathway, three genes—B2M (Beta-2-Microglobulin), CIP2A (Cancerous Inhibitor of PP2A), and BCL2L1 (B-cell lymphoma 2-like 1)—exhibited elevated expression in tumors from Black patients and were also linked to markedly poorer outcomes, with hazard ratios exceeding 2 and reductions in median survival by 49.6, 5.6, and 21.7 months, respectively. Additionally, lower expression levels of the immune-related genes IL2 (Interleukin 2) and FOXP3 (forkhead box 3) were associated with reduced median survival by 11.5 and 13.4 months, respectively.

These findings suggest that differential gene expression may represent a biological factor contributing to the observed disparities in disease progression and survival outcomes between Black and White patients with PDAC. In summary, differences in the incidence and clinical course of PDAC among Black patients may arise not only from environmental and lifestyle-related factors but also from distinct gene-expression profiles. Molecular alterations such as TSPAN8 overexpression or dysregulation of PD-1/PD-L1 pathway related genes may contribute to poorer treatment outcomes and reduced survival in this population.

#### 3.2.3. Sex

Some available studies in prognostic analyses have shown that female gender may be a favorable prognostic factor in PDAC patients. Fard et al. [13], in their meta-analysis, indicated a significant association between patient gender and overall survival, with women achieving better survival outcomes than men (HR = 1.182, 95% CI [1.083, 1.291], *p* < 0.001). This may be due to the fact that women are more likely to take care of their health and utilize healthcare services, which allows for earlier detection and initiation of appropriate treatment [5].

On the other hand, Gehrels et al. [59] observed that women with stage I-III pancreatic cancer received systemic chemotherapy less frequently than men. These differences are particularly noticeable in the oldest age group, where nearly half of the patients received only best supportive care (BSC). One possible explanation for this trend is that female patients were generally older and likely presented with poorer health status. Women may also be stereotypically perceived as weaker, which could reduce their chances of receiving more invasive treatments. Furthermore, when comparing all patients receiving systemic, surgical, and BSC treatment, it was found that women had a shorter overall survival time compared to men (with mOS of 8.5 and 9.2 months, respectively). Stratified by age, women were significantly more likely to receive only BSC in the oldest age group (82.4% vs. 89.5%, *p* < 0.001). The main reason for choosing BSC over surgery among the male population was clinical factors related to the patient’s condition, whereas treatment preferences more frequently played a decisive role in women. Interestingly, higher overall survival (OS) values were observed in the female cohort, although the difference did not reach statistical significance. It should also be emphasized that these data were collected retrospectively from physicians’ notes in medical records and therefore reflect the interpretation of the medical staff rather than direct patient-reported preferences.

PDAC is associated with higher incidence and mortality among men compared to women. The incidence of PDAC among men is higher than in women, as well as mortality, based on data from the International Agency for Research on Cancer (IARC) in 2022 [60]. These differences can be attributed to various factors, including genetic, physiological, and environmental influences. Men are more frequently exposed to risk factors such as smoking, excessive alcohol consumption, and obesity, which increase the risk of pancreatic cancer. The role of estrogen signaling in anti-tumor immunology is still not completely understood in the context of cancers unrelated to the reproductive system, such as PDAC. However, estrogen receptors in PDAC cells may play a prognostic role. These receptors may have a protective function in promoting antitumor immune responses [61,62,63].

In the study conducted by Zou et al. [61], immunohistochemical (IHC) staining was used to identify expression patterns of three estrogen receptors (e.g., estrogen receptor alpha (ERα), estrogen receptor beta (ERβ), and G protein-coupled estrogen receptor (GPER) in 174 postsurgical specimen from patients with PDAC. The expression of estrogen receptors with transcriptomic RNA-Seq analysis and IHC staining was correlated to the presence of tertiary lymphoid structures (TLS). TLS are ectopic aggregates of immune cells that arise within the tumor microenvironment in response to inflammation and function similarly to lymph nodes—they serve as local sites for the activation, differentiation, and interaction of T and B lymphocytes as well as antigen-presenting cells. The study confirmed that TLS presence correlates with increased infiltration of activated CD8+ and CD4+ T cells, along with elevated expression of estrogen receptors (ERα, ERβ, and GPER). The expression of estrogen receptors, particularly ERα and ERβ, was not only associated with the formation of TLS but also enhanced CD8+ T cell chemotaxis toward cancer cells in vitro, indicating their role in promoting antitumor immune responses. These findings suggest that estrogens, through their receptors, may support TLS development and local immune activation, contributing to a more favorable clinical course of PDAC. Notably, the best prognosis was observed in female patients with TLS (mOS—3 years), while the worst outcomes were seen in male patients lacking TLS (mOS—2 years), highlighting a potential link between estrogen signaling, immune response, and prognosis in PDAC [64].

Moreover, Andersson et al. [64] analyzed the association between hormonal and reproductive factors and the risk of PDAC in women. The analysis included 17,035 women aged 44 to 73 years, who, between 1991 and 1996, completed detailed questionnaires on lifestyle, health status, use of oral contraceptives (OC), and hormone replacement therapy (HRT). Incident cases of PDAC were identified through the Swedish Cancer Registry up to December 31, 2015, resulting in a total of 110 PDAC cases. Statistical analysis was performed using Cox proportional-hazards models, adjusted for age and known risk factors such as smoking, body mass index (BMI), and alcohol consumption. HRT was associated with a significantly reduced risk (fully adjusted HR = 0.48; 95% CI: 0.23–1.00), with an even stronger protective effect observed for estrogen-only HRT (HR = 0.22; 95% CI: 0.05–0.90).

Based on these findings, the authors concluded that exposure to estrogens may play a protective role in the development of PDAC in women. Estrogen-based HRT, in particular, may exert beneficial effects by influencing the PDAC microenvironment, regulating cell proliferation, and enhancing immune defense mechanisms [61]. Activation of ERα by estradiol leads to the modulation of key signaling pathways, including MAPK/ERK (Mitogen-Activated Protein Kinase/Extracellular Signal-Regulated Kinase), which under physiological conditions regulate cell cycle progression and proliferation. Preclinical studies have demonstrated that ERα stimulation results in reduced ERK1/2 (Extracellular signal-Regulated Kinases 1 and 2) activity, thereby decreasing tumor cell proliferation and slowing tumor progression [65].

Female sex appears to be modestly protective, possibly owing to estrogen-mediated immune activation and higher rates of intratumoral lymphoid structures. Nevertheless, treatment disparities—women being offered systemic therapy less often—may counterbalance this advantage. Hormonal influences, including estrogen receptor expression and hormone replacement therapy, are increasingly investigated as modulators of prognosis.

#### 3.2.4. Nutritional State

In patients with PDAC, limited oral intake, loss of appetite, catabolic processes induced by the presence of a malignant tumor, and reduced intestinal absorption due to obstruction of the main pancreatic duct or exocrine pancreatic insufficiency can negatively affect the nutritional status, leading to malnutrition and muscle mass loss [66]. Decreased muscle mass in cancer patients before planned pancreatic resection is correlated with poorer long-term survival rates [67,68].

Malnutrition and sarcopenia are common issues in patients with PDAC are associated with an increased risk of chemotherapy-related toxicity and reduced survival (mOS sarcopenic 17.7 months vs. non-sarcopenic 33.2 months) [68]. More than half of PDAC patients experience weight loss at the time of diagnosis, and most develop malnutrition during chemotherapy treatment [69,70].

The study conducted by Lu et al. [71] aimed to evaluate the impact of preoperative nutritional status on the survival of patients with PDAC who underwent surgical resection, using the prognostic nutritional index. PNI is calculated based on the serum albumin level and the lymphocyte count according to the formula: PNI = (10 × serum albumin level (g/dL)) + (0.005 × lymphocyte count). Patients with a low PNI (below 47.30) had a median OS of 19.47 months, while those with a high PNI (47.30 or above) had a median OS of 46.30 months, which was statistically significant (*p* < 0.0001).

Sarcopenia and cachexia are wasting syndromes that lead to profound disturbances in overall physiological function. Their progression is associated with the loss of skeletal muscle and adipose tissue, depletion of energy reserves, and impairment of the immune system, which significantly increases susceptibility to infections and reduces the patient’s ability to tolerate oncological treatment. In advanced stages of cachexia, the wasting process affects not only muscle and fat tissue but also leads to the dysfunction of multiple organs including the heart, liver, brain, intestines, and hypothalamus, disrupting the body’s metabolic and hormonal balance. Loss of muscle mass contributes to disturbances in protein and energy metabolism. These alterations, coupled with the activation of catabolic pathways (e.g., mediated by inflammatory cytokines and the stimulation of brown adipose tissue), result in rapid deterioration of the patient’s general condition [72]. The prognosis for patients with PDAC who develop irreversible cancer cachexia is unfavorable, with an expected survival time of less than 3 months [66]. An analysis by Menozzi et al. [73] demonstrated that a lower body mass index (BMI) and weight loss are significantly associated with a higher risk of postoperative complications classified as grades I-II on the Clavien scale. This scale is a classification system used to assess and categorize postoperative complications based on their severity. Additionally, reduced muscle mass before surgery was identified as an independent predictor of an increased risk of gastrointestinal hemorrhage after surgery [69,73].

An important issue is the exocrine pancreatic insufficiency (PEI), which affects 72% of patients with advanced PDAC. Unfortunately, this is often unrecognized; the symptoms are attributed to PDAC itself and the targeted treatment is omitted. The most common symptoms of PEI are steatorrhea, weight loss even with preserved appetite, diarrhea, bloating, and abdominal pain. Patients may also experience deficiencies in fat-soluble vitamins, leading to osteoporosis. The diagnosis of PEI is usually based on the measurement of fecal elastase-1 (FE-1). Low levels of elastase (<200 µg/g of stool) suggest the need for pancreatic enzyme replacement therapy (PERT). PERT involves administering pancreatic lipase in capsules. This therapy is highly effective in alleviating the symptoms of PEI, such as fatty stools and weight loss, and improves nutrient absorption. Additionally, dietary support is recommended, including a diet rich in protein and calories, as well as supplementation with fat-soluble vitamins. Early recognition and treatment of PEI are crucial, as they improve nutritional status, quality of life, and may increase the survival of PDAC patients by 3.8 months [74,75,76].

Based on the above paragraph, it should be highlighted that malnutrition, sarcopenia, and cachexia are common and significantly worsen patient outcomes. Reduced oral intake, tumor-induced catabolism, and PEI contribute to muscle and fat loss, impaired immune function, and organ dysfunction, increasing susceptibility to complications and reducing survival. Preoperative nutritional status, measured by indices like PNI, strongly predicts survival, while low BMI and weight loss raise the risk of postoperative complications.

#### 3.2.5. Smoking Cigarettes

Results from large cohort analyses indicate that cigarette smoking has significant prognostic implications in patients with PDAC. In a study conducted at the China National Cancer Center, which included over 1700 patients with histologically or cytologically confirmed diagnoses, marked survival differences were observed depending on smoking history. Never-smokers had a median overall survival of approximately 8.5 months, whereas current smokers demonstrated a median survival of less than 8 months, and former smokers slightly above 7 months. The duration of exposure proved critical: patients with a smoking history of at least three decades had a median survival of only 6.5 months, representing a substantially worse prognosis compared with never-smokers. In the unresectable disease setting, heavy smoking (≥20 cigarettes per day) was associated with more than a twofold increase in the risk of death [77].

Similar conclusions emerged from prospective United States of America analyses, encompassing more than one thousand patients drawn from two long-term observational cohorts, as well as an additional subgroup with available biospecimens. Smoking status was determined both via standardized questionnaires and by measurement of plasma cotinine concentrations—a nicotine metabolite—using liquid chromatography–tandem mass spectrometry (LC-MS/MS). Current smokers at the time of diagnosis exhibited approximately one-third poorer survival outcomes compared with never-smokers, with even higher mortality risk among those with substantial cumulative exposure (>60 pack-years). In the biomarker analysis, elevated plasma cotinine concentrations were likewise associated with markedly reduced survival: in certain subgroups, median survival was only 4 months, compared with 7 months in patients without detectable exposure [78].

Despite methodological differences, both studies draw the same conclusion: long-term and intensive tobacco use constitutes an independent adverse prognostic factor in PDAC. Moreover, while smoking cessation may confer partial benefit, the evidence suggests that prolonged exposure leaves a lasting negative impact on patient outcomes.

#### 3.2.6. Alcohol Intake

In a study including 1783 patients with PDAC, alcohol consumption prior to diagnosis was shown to be associated with poorer prognosis. The mOS in the entire cohort was 8.3 months, with a five-year survival rate of only 4.2%. Among never-drinkers (77.2% of patients), the median OS reached 8.8 months, whereas in drinkers (22.3%) it was shorter at 6.5 months. The most unfavorable outcomes were observed in individuals consuming ≥53.5 g of ethanol per day (classified as heavy drinking). In this group, the mOS was 6.4 months, and the risk of death was 45% higher compared with abstainers (HR 1.45; 95% CI 1.03–2.05). A similar effect was observed among patients with metastatic disease (approximately 70% of the entire cohort), where in the same consumption category the risk of death increased by 55% (HR 1.55; 95% CI 1.01–2.38). Alarmingly, patients who combined alcohol intake with tobacco smoking also demonstrated worse outcomes: current smokers and drinkers had a median OS of 6.4 months, compared with 8.8 months among never-smokers and abstainers [77].

These findings are consistent with data from a large meta-analysis of 30 prospective cohorts (2,494,432 participants, 10,067 pancreatic cancer cases), which demonstrated that each additional 10 g/day of alcohol intake increased the risk of pancreatic cancer by 3% (HR 1.03; 95% CI 1.02–1.04). Compared with individuals with very low consumption (0.1–5 g/day), the risk was 12% higher among those consuming 30–60 g/day (HR 1.12; 95% CI 1.03–1.21), and as much as 32% higher with an intake of ≥60 g/day (HR 1.32; 95% CI 1.18–1.47). In women, an elevated risk was observed starting at 15–30 g/day (12% increase), whereas in men the threshold was ≥30 g/day (15% increase for 30–60 g/day and 36% increase for ≥60 g/day) [79].

#### 3.2.7. Sedentarism and Physical Activity

Based on epidemiological data and translational studies, sedentary behavior and low levels of physical activity have been identified as adverse prognostic factors in PDAC. Prospective cohort studies demonstrated that individuals meeting the criteria for moderate physical activity (42–62.9 MET hours/week) exhibited a significantly lower risk of PDAC (HR = 0.59) compared with sedentary populations [80].

Similarly, interventional studies involving patients undergoing systemic therapy or following tumor resection (sample sizes ranging from 30 to 317 participants) showed that increased physical activity correlated with reduced fatigue severity, improved health-related quality of life (HRQoL; β = 0.03; *p* = 0.02), and better treatment tolerance, whereas predominant sedentary behavior was associated with deterioration in quality-of-life parameters (β = −0.02; *p* = 0.01) [80].

These observations are corroborated by preclinical models. In murine studies with KrasG12D-driven (Kristen Rat Sarcoma viral oncogene homolog) pancreatic carcinogenesis and diet-induced obesity (DIO), exercise intervention in the form of voluntary wheel running (average 4–5 km/day; DIO + PA group, diet-induced obesity + physical activity) significantly reduced weight gain (*p* = 0.020), decreased pancreatic inflammation and fibrosis (*p* = 0.001), delayed progression of PanIN, and completely prevented PDAC development (43% incidence in DIO vs. 0% in DIO + PA) [81].

The mechanisms underlying these effects include reduction in obesity and improvement of metabolic regulation (lower glycemia and enhanced insulin sensitivity), anti-inflammatory activity (reduced levels of proinflammatory cytokines such as IL-6 (Interleukin-6), TNF- α (Tumor Necrosis Factor α), IL-1β (Interleukin-1β), and MCP-1 (Monocyte Chemoattractant Protein-1)), attenuation of pancreatic fibrosis and PanIN burden, improved tumor vascularization and reduced hypoxia (enhancing systemic therapy efficacy), as well as modulation of adipokine signaling, including increased expression of the IL-15ra (Interleukin-15 receptor α) receptor in adipose tissue. Experimental models further showed that IL-15 (Interleukin-15) overexpression slowed PDAC growth, particularly in non-obese subjects [81].

Taken together, the accumulated quantitative and qualitative evidence indicates that physical activity exerts a dual role—reducing the risk of PDAC in high-risk populations and improving clinical and prognostic outcomes among patients with established disease—whereas sedentary behavior remains a negative determinant [80,81].

#### 3.2.8. Obstructive Sleep Apnea (OSA)

In studies conducted by Shoucair et al. [82], obstructive sleep apnea (OSA) was identified as a significant prognostic factor in patients with pancreatic cancer, particularly in the context of PDAC. In a retrospective study including 334 PDAC patients who underwent neoadjuvant therapy (NAT) followed by surgical resection, OSA was shown to be an independent predictor of poor histopathological response to treatment (OR 2.72; 95% CI: 1.35–5.49; *p* = 0.005). Patients received contemporary multi-agent NAT regimens—either chemotherapy alone or combined chemoradiotherapy, with the latter being associated with a more favorable pathological response (OR 0.39; 95% CI: 0.19–0.79; *p* = 0.008). Treatment response was assessed according to the College of American Pathologists (CAP) tumor regression grading system, with favorable response defined as CAP 0–1 and unfavorable response as CAP 2–3. Histopathological evaluation was performed on postoperative specimens in line with current grading standards. Patients with OSA were more likely to present with larger tumors, vascular and perineural invasion, and shorter durations of neoadjuvant treatment. The mOS in this cohort was 27.3 months; however, patients with OSA had significantly shorter OS compared with those without the disorder (20.4 vs. 30.5 months; *p* = 0.009). One- and three-year survival rates in the OSA group were 68.4% and 28.9%, respectively, compared with 79.7% and 45.5% in the control group.

In a study by Song et al. [83], involving 200 elderly patients with stage I pancreatic cancer who underwent Whipple resection followed by chemotherapy, the impact of sleep quality was evaluated both objectively (using wearable devices) and subjectively. Subjective assessment utilized the Richards–Campbell Sleep Questionnaire (RCSQ)—a five-item visual analog scale measuring in-hospital sleep quality—and the Pittsburgh Sleep Quality Index (PSQI)—a multidimensional instrument assessing sleep quality over a one-month period. Patients were stratified into two prognostic groups: favorable (n = 106) and unfavorable (n = 94), based on the occurrence of postoperative complications such as deep infections, fistula, reoperation, respiratory failure, or sepsis. Reduced sleep efficiency emerged as one of the strongest independent prognostic factors—each 1% decrease in efficiency was associated with a more than 26-fold increased risk of unfavorable outcomes (OR 26.595; *p* < 0.001). Moreover, patients with better sleep quality demonstrated significantly longer median OS (24.05 vs. 20.91 months; *p* = 0.002) and prolonged progression-free survival (PFS) (7.06 vs. 6.08 months; *p* = 0.001). Multivariate analyses confirmed that sleep parameters influenced both postoperative complication risk and long-term treatment outcomes.

In summary, both clinically diagnosed OSA and objectively reduced sleep efficiency may represent independent prognostic factors in patients with PDAC. Sleep-disordered breathing and impaired sleep quality are associated with poorer response to neoadjuvant therapy, greater tumor aggressiveness, and significantly shorter OS and PFS, suggesting their potential utility as prognostic markers in risk stratification and therapeutic decision-making.

#### 3.2.9. Psychological Factors

In the cross-sectional study by Clark et al. [84], including 304 patients with PDAC and 7749 patients with other malignancies, psychological distress was assessed using the Brief Symptom Inventory (BSI/BSI-18) and the Problem Common Checklist (PCL). High distress was defined as a T-score ≥63 on the Global Severity Index (GSI) or on at least two BSI subscales; in the BSI-18, a threshold of ≥10 for men and ≥13 for women was applied (the T-score is a standardized score with a population mean of 50 and a standard deviation of 10; values ≥ 63 indicate clinically significant symptom severity). Patients with PDAC more frequently reported severe symptoms of depression (28.8% vs. 18.5%), anxiety (29.1% vs. 23.9%), somatization (33.8% vs. 28.3%), and global distress (26.4% vs. 21.0%) compared to patients with other cancers. Notably, within this group, men reported depressive symptoms significantly more often than women (34.0% vs. 22.6%), which represents a reversal of the typical pattern observed in the general population. The authors suggested that this finding may be attributable to both biological mechanisms (e.g., the role of proinflammatory cytokines such as IL-6, which induce depressive symptoms) and psychosocial factors (men are less likely to seek psychological support and tend to prefer problem-focused coping strategies, which may promote the accumulation of symptoms). The authors concluded that routine distress screening, along with psychosocial and pharmacological interventions, is necessary to improve both quality of life and treatment adherence in this patient population.

In the study conducted by Ebstein et al. [85], the aim was to analyze the experience of psychological stress in patients with PDAC across all stages of the disease. The authors emphasized that this malignancy is associated with the highest level of emotional burden among cancers, further compounding its poor prognosis (5-year survival approximately 19%, 10-year survival approximately 10%). In this patient group, stress is subjective and multidimensional, encompassing emotions related to diagnosis, concerns about treatment course, financial burden, changes in family relationships, and fear of death. Biologically, chronic stress activates the HPA axis (Hypothalamic–Pituitary–Adrenal axis) and sympathetic nervous system, leading to the release of glucocorticoids, catecholamines, and proinflammatory cytokines (e.g., IL-6), which disrupt tryptophan–kynurenine, glutamatergic, and serotonergic pathways. Clinically, this manifests as depression, fatigue, sleep disturbances, impaired concentration, and exacerbation of pain. In a 19-year cohort study including 4363 individuals, two-thirds of patients reported psychological stress, which was associated with increased cancer-specific mortality, including pancreatic cancer. The authors concluded that systematic identification and reduction in stress in PDAC patients—through education, psychosocial support, and interventions aimed at enhancing adaptive coping—are essential not only for alleviating psychological suffering but also for potentially improving treatment outcomes.

## 4. Conclusions

**Figure 2:**
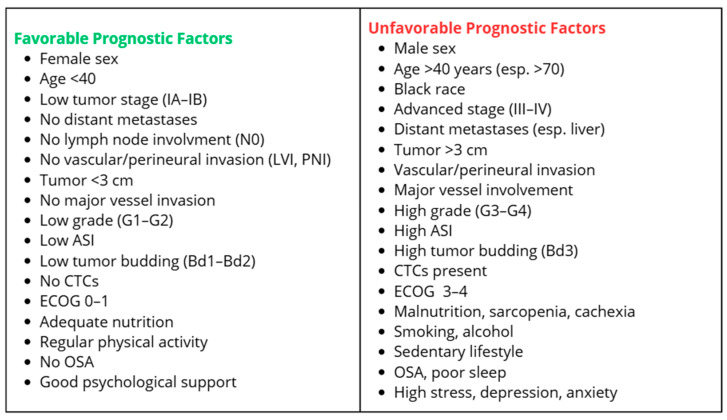
Prognostic map of Pancreatic ductal adenocarcinoma (PDAC).

PDAC is one of the most aggressive malignancies and is characterized by an exceptionally poor prognosis. Prognostic factors for PDAC can be categorized into favorable and unfavorable ones. For the purpose of systematizing the current knowledge, they have been organized in the diagram below (Figure 2).

Classical prognostic factors such as TNM stage, tumor size, presence of metastases, and lymph node involvement are well established and widely used in clinical practice. However, it is important to emphasize the growing significance of additional, less commonly applied factors, which may in the future substantially influence patient risk stratification.

Of particular interest is the prognostic difference between women and men. Data indicate that estrogens—through their effects on the tumor microenvironment, the presence of estrogen receptors, and the formation of intratumoral lymphoid structures—may promote local immune activation and improve survival in female patients. By contrast, men, who more frequently carry risk factors such as tobacco use, alcohol consumption, and obesity, demonstrate worse outcomes.

Another important aspect is the role of desmoplasia. Stromal expansion is associated with poor prognosis; however, novel diagnostic techniques such as EUS remain underutilized despite offering noninvasive differentiation of lesions and precise assessment of tissue stiffness. This approach may hold clinical relevance in the early detection and characterization of PDAC.

Tumor budding and CTCs represent emerging but still underexplored prognostic markers in PDAC. Tumor budding reflects histologic invasiveness, while CTCs indicate the risk of dissemination and recurrence even in the absence of radiologically detectable metastases. Although both phenomena are well recognized in other cancers, they remain underappreciated in PDAC, yet may in the future support treatment personalization and earlier detection of disease progression, highlighting the need for further investigation.

A frequently overlooked but clinically important issue is exocrine pancreatic insufficiency (PEI), which affects the majority of patients with advanced PDAC. Its symptoms—such as steatorrhea, weight loss despite preserved appetite, and vitamin deficiencies—are often misattributed to the cancer itself, delaying enzyme replacement therapy. Pancreatic enzyme replacement therapy (PERT) improves nutrient absorption, nutritional status, and quality of life, and may also prolong survival. In the context of cancer-associated wasting and cachexia, management of PEI therefore has significant prognostic implications that remain underrecognized in daily practice.

In summary, prognostic assessment in PDAC should extend beyond classical TNM criteria. Sex-related differences and the role of estrogens, the use of EUS elastography in evaluating desmoplasia, novel biological markers such as CTCs and tumor budding, as well as the early recognition and treatment of PEI, are examples of factors that could substantially improve prognostication and therapeutic strategies in patients with PDAC.

## Figures and Tables

**Table 1:** Median survival time of patients (months) by stage of pancreatic cancer according to the UICC (Union for International Cancer Control) and AJCC (American Joint Committee on Cancer) [6,7,8].

Disease Stage (TNM)	Median Overall Survival (mOS) [Months]
IA	29.0–125.9
IB	22.0–36.0
IIA	20.0–38.1
IIB	16.0–24.4
III	16.0–16.6
IV	9.0–10.6

**Table 2:** Survival duration (months) among patients with/without vascular invasion. CA—celiac artery; CHA—common hepatic artery; PV—portal vein; SMA—superior mesenteric artery; SMV—superior mesenteric vein [14,15].

Involved Vessel	No Contact/Invasion [Months]	Invasion < 180° [Months]	Invasion ≥ 180° [Months]
SMV/PV	29.6–34.2	21.7–29.7	17.3–20.0
SMA	27.6–34.2	14.3–19.3	15.8–17.4
CA	26.4–34.2	14.3–18.1	15.8–17.1
CHA	25.5–34.2	14.3–22.8	15.8–16.5

**Table 3:** Median overall survival (mOS) based on lymph node involvement parameters in PDAC.

Category	Subgroup	Median OS [Months]
Lymph Node Ratio (LNR)	0 (no positive nodes)	25
	>0–0.2	22
	>0.2–0.4	15
	>0.4	12

**Table 4:** ECOG scale. The table shows data on OS and recommended treatment in patients with PDAC according to PS. OS—overall survival [45,46,47].

ECOG	Characteristics of the Patient	OS [Months]	Recommended Treatment
0	Fully active, able to carry on all predisease performance without restriction	~20 (17.1–22.9)	FOLFIRINOX or Gemcitabine with NAB-paclitaxel
1	Restricted in physically strenuous activity but ambulatory and able to carry out work of a light or sedentary nature (e.g., light housework, office work)	~10.7 (9.5–11.9)	FOLFIRINOX or Gemcitabine with NAB-paclitaxel
2	Ambulatory and capable of all self-care but unable to carry out any work activities; up and about more than 50% of waking hours	~6.2 (4.8–7.6)	Gemcitabine as monotherapy
3	Capable of only limited self-care; confined to bed or chair more than 50% of waking hours	~3.8 (1–6.6)	Cancer therapy only on a case-by-case basis. Optimization of supportive care measures.
4	Completely disabled; cannot carry out any self-care; totally confined to bed or chair	~3.8 (1–6.6)	Cancer therapy only on a case-by-case basis. Optimization of supportive care measures.

**Table 5:** The median survival time in different age groups with PDAC [49,50,51].

Age	Median Survival Time [Months]
20–40	36
40–60	10.0–10.4
60–70	8.0–23.8
70–80	8.0–17.3
>80	4.0–6.4

**Table 6:** Summary of the major studies aimed at evaluating the impact of age on the prognosis of patients with PDAC.

Author (Year)	Population Size	Age Range	Results	Conclusion
Amin et al. (2013) [50]	45,509 patients	<50 to >70 y.o.	OS was negatively correlated with age (<50 y.o. = 10.4 months; 50–70 y.o. = 9.1 months; >70 y.o. = 6.4 months).	Younger patients had a better prognosis, reflected by higher OS.
Wang et al. (2020) [49]	126,066 patients	20 to >80 y.o.	The 5-year survival for patients with PDAC aged 20–40 years was approximately three times higher compared to patients over 40 years of age.	Older age is associated with shorter survival.
Tao et al. (2021) [5]	10,784 patients	<65 to 80 y.o.	CSS:	The older the patients, the higher overall mortality, but the lower the cancer-specific mortality.
			<65 y.o. = 82.8%;	
			65–80 y.o. = 80.8%;	
			≥80 y.o. = 76.8%	
			OS:	
			<65 y.o. = 91.7%;	
			65–80 y.o. = 95.2%;	
			≥80 y.o. = 97.1%;	
Hackner et al. (2022) [51]	213 patients	<70 vs. ≥70 y.o.	mOS was significantly shorter in patients ≥70 years compared to those <70 years (17.1 vs. 29.2 months).	Age is an important prognostic factor in patients with PDAC who underwent primary radical resection.

CSS—cancer-specific survival; mOS—median overall survival; OS—overall survival; PDAC—pancreatic ductal adenocarcinoma; y.o.—years old.

## Data Availability

Not applicable.
